# Supplementary figures and images for: Expanding the tolerance of segmented Influenza A Virus genome using a balance compensation strategy
Source: PLoS Pathog. 2022 Aug 4;18(8):e1010756. doi: 10.1371/journal.ppat.1010756 (PMC9380948; doi:10.1371/journal.ppat.1010756)

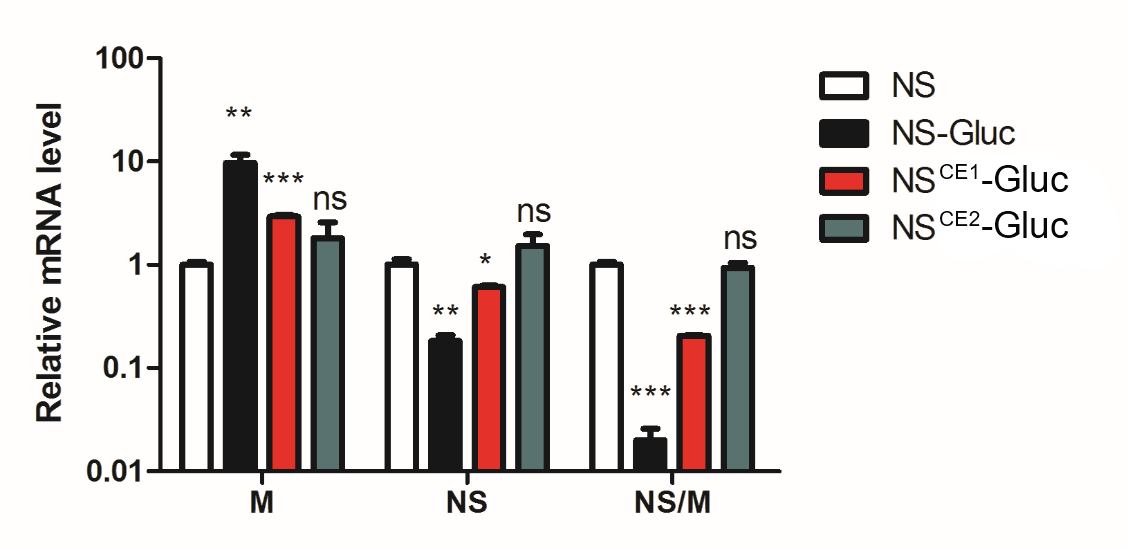

Supplement: S1 Fig — The indicated NS-derived vRNAs were separately subjected to dual competition assay with wildtype M as competitive control. Data represents the relative mRNA levels of wildtype M, NS and normalized NS/M. *, p<0.05; **, p<0.01; ***, p<0.001; ns, no significance; students’ t test. (TIF) [file ppat.1010756.s001.tif]

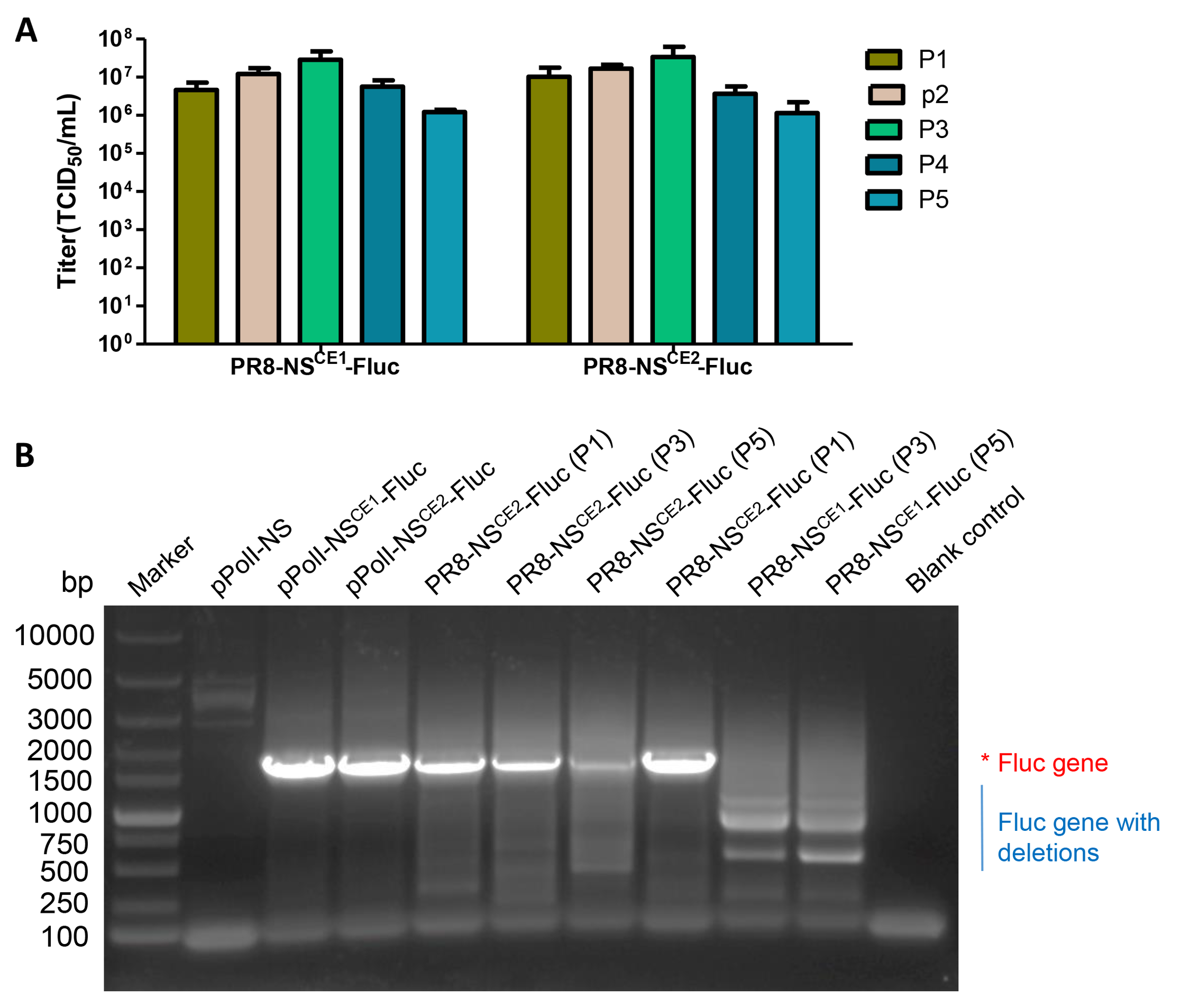

Supplement: S2 Fig — (A) The indicated reporter viruses were serially passaged in chicken embryos and the titer of each passage was determined. Error bars indicated Mean ± SEM of three independent experiments. (B) The genome RNAs of indicated recombinant viruses were extracted using TIANamp Virus RNA Kit (Tiangen, China). The complementary DNA was prepared using PrimeScript RT reagent Kit with gDNA Eraser (Takara, China) and NS segment specific primer (5’-CAGGGTGACAAAGACATAATG-3’). Then PCR analysis was performed using the 2xTaq MasterMix (Cwbio, China) and primers covering the full length of firefly luciferase gene (NS-Fluc-specific-Forward:5’-ACGTCGAGGAGAATCCCGGGCCCATGGAAGACGCCAAAAA-3’; NS-Fluc-specific-Reverse: CAGGCTAAAGTTGGTCGCGCCGCTGCCCAATTTGGACTTT). The PCR product was analyzed using 1% agarose gel electrophoresis. The plasmids pPolI-NSCE1-Fluc and pPolI-NSCE2-Fluc were used as positive controls, while pPolI-NS was used as the negative control. (TIF) [file ppat.1010756.s002.tif]

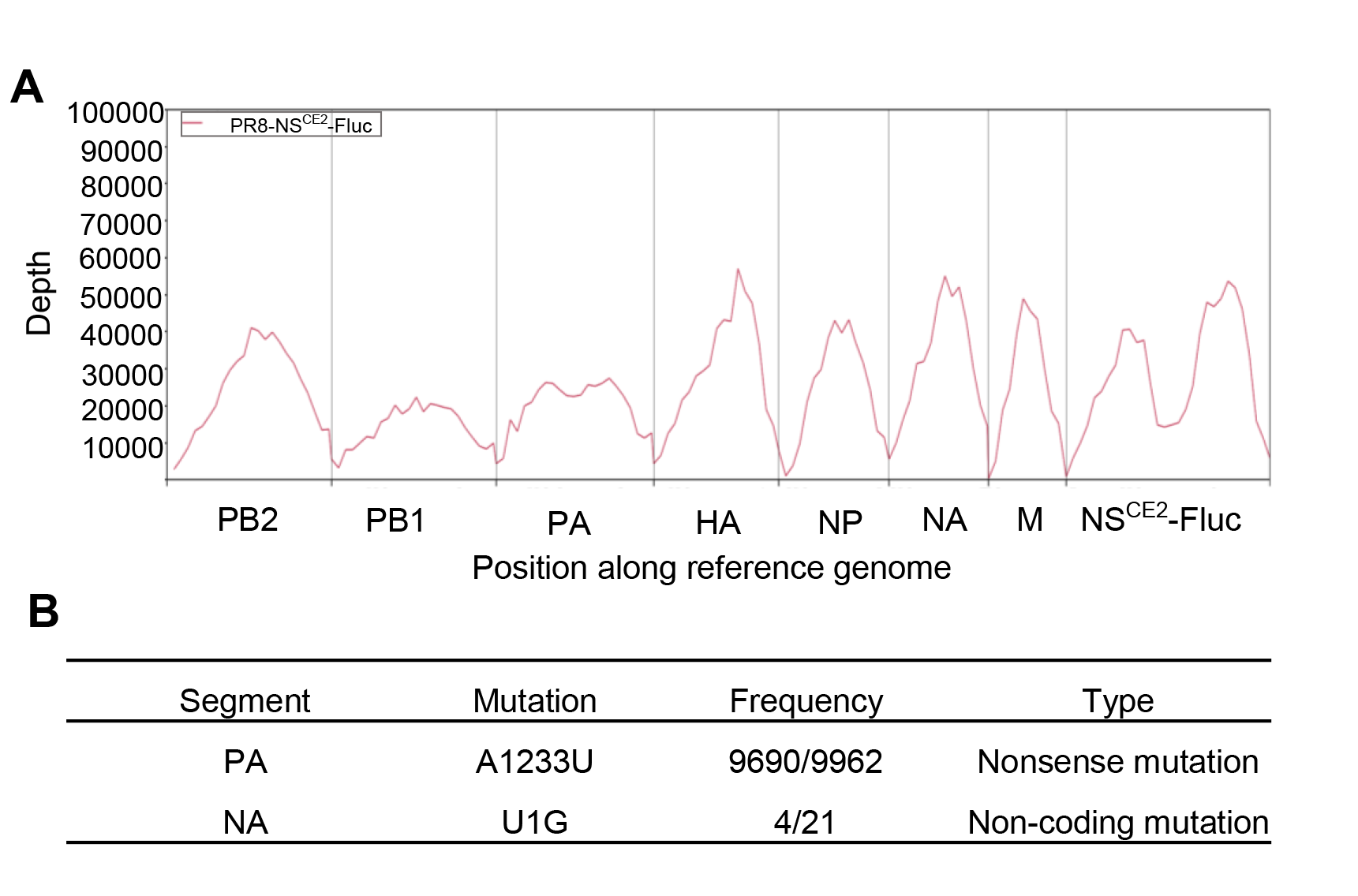

Supplement: S3 Fig — The viral RNA of PR8-NSCE2-Fluc (Passage 5) was extracted using SparkZol reagent (SparkJade, China) according to the manufacturers manual and sequentially subjected to first and second strand cDNA synthesis using BeyoRT II First Strand cDNA Synthesis Kit (RNase H-) and Second Strand cDNA Synthesis Kit (Beyotime, China). The double stranded cDNA library of virus genome was deep sequenced by Novogene (China) using Illumine nova 6000. Structural variation (SV) analysis by BreakDancer software (V1.4.4, http://breakdancer.sourceforge.net/) detected no insertion, deletion, inversion and translocation of the large segments in the genome level, While SNP/InDel analysis using SAMTOOLS identified two substitutions but no insertion/deletion. (A) The depth of sequencing to positions along the reference genome. (B) Identification and characterization of the mutations. (TIF) [file ppat.1010756.s003.tif]
