# Supplementary material for: Expanding the tolerance of segmented Influenza A Virus genome using a balance compensation strategy
Source: PLoS Pathog. 2022 Aug 4;18(8):e1010756. doi: 10.1371/journal.ppat.1010756 (PMC9380948; doi:10.1371/journal.ppat.1010756)
Supplement: S1 Table — a The substitutions at indicated positions are shown in red. (DOCX) [file ppat.1010756.s004.docx]

**S1 Table.** Primers for construction of the plasmids.

| Primer | Sequences (5’-3’) ^a^ |
| --- | --- |
| NCR_NS_-Fluc-Forward | GGGACCATGCCGGCCAGCAAAAGCAGGGTGACAAAGACATAATGGAAGACGCCAAAAACATAAAG |
| NCR_NS_-Fluc-Reverse | GGGCCGCCGGGTTATTAGTAGAAACAAGGGTGTTTTTTATTATTACAATTTGGACTTTCCGCCCTTC |
| NCR_NS_-Rluc-Forward | GGGACCATGCCGGCCAGCAAAAGCAGGGTGACAAAGACATAATGACTTCGAAAGTTTATGATC |
| NCR_NS_-Rluc-Reverse | GGGCCGCCGGGTTATTAGTAGAAACAAGGGTGTTTTTTATTATTATTGTTCATTTTTGAGAACTC |
| NCR_NS_-3m-Forward | GGGACCATGCCGGCCAGTAAAAACGGGGTGACAAAGACATAATG |
| NCR_NS_-5m-Forward | GGGACCATGCCGGCCAGCAGAAGCAGGGTGACAAAGACATAATG |
| NCR_NS_-8m-Forward | GGGACCATGCCGGCCAGCAAAAACAGGGTGACAAAGACATAATG |
| NCR_NS_-3,5m-Forward | GGGACCATGCCGGCCAGTAGAAGCAGGGTGACAAAGACATAATG |
| NCR_NS_-3,8m-Forward | GGGACCATGCCGGCCAGTAAAAACAGGGTGACAAAGACATAATG |
| NCR_NS_-5,8m-Forward | GGGACCATGCCGGCCAGCAGAAACAGGGTGACAAAGACATAATG |
| NCR_NS_-3,5,8m-Forward | GGGACCATGCCGGCCAGTAGAAACAGGGTGACAAAGACATAATG |
| 3’-NCR-Forward | GGGACCATGCCGGCCAGCAAAAGCAGG |
| NS1-2A-Reverse | CGTCCCCGGCTTGCTTGAGGAGAGAGAAATTTGTGGCTCCGGACCCAACTTCGCTTCT |
| 2A-Fluc-Forward | CAAGCAAGCCGGGGACGTCGAGGAGAATCCCGGGCCCATGGAAGACGCCAAAAACATA |
| Fluc-2A-Reverse | GTCGCGCCGCTGCCCAATTTGGACTTTCCGCCCTTC |
| 2A-NEP-Forward | GTCCAAATTGGGCAGCGGCGCGACCAACTTTAG |
| 5'-NCR-Reverse | GGGCCGCCGGGTTATTAGTAGAAACAAGG |

^a^ The substitutions at indicated positions are shown in red.
