# Supplementary material for: Expanding the tolerance of segmented Influenza A Virus genome using a balance compensation strategy
Source: PLoS Pathog. 2022 Aug 4;18(8):e1010756. doi: 10.1371/journal.ppat.1010756 (PMC9380948; doi:10.1371/journal.ppat.1010756)
Supplement: S2 Table — (DOCX) [file ppat.1010756.s005.docx]

**S2 Table.** Primers for reverse transcription and qPCR analysis.

| Primer | Sequence（5’-3’） |
| --- | --- |
| RT-vRNA-NS | GATCGCTCTTCTGGGAGCAAAAGCAGG |
| RT-3’NCR | CAGGGTGACAAAGACATAATG |
| qPCR-Rluc-Forward | GGAATTATAATGCTTATCTACGTGC |
| qPCR-Rluc-Reverse | CTTGCGAAAAATGAAGACCTTTTAC |
| qPCR-Fluc-Forward | GGATCTACTGGGTTACCTAAGG |
| qPCR-Fluc-Reverse | GGGTTGGTACTAGCAACGCAC |
| qPCR-NS-Forward | ATTTCACCATTGCCTTCT |
| qPCR-NS-Reverse | GGTCTCCCATTCTCATTAC |
| qPCR-M-Forward | CTTCTAACCGAGGTCGAAAC |
| qPCR-M-Reverse | CGTCTACGCTGCAGTCCTC |
