# Supplementary material for: Expanding the tolerance of segmented Influenza A Virus genome using a balance compensation strategy
Source: PLoS Pathog. 2022 Aug 4;18(8):e1010756. doi: 10.1371/journal.ppat.1010756 (PMC9380948; doi:10.1371/journal.ppat.1010756)
Supplement: Attachment — Submitted filename: Comments_PLOS Pathogens Expanding the Tolerance of Segmented Influenza A Virus Genome Using a Balance Compensation Strategy.docx [file ppat.1010756.s006.docx]

The work by Zhao and collaborators (Expanding the Tolerance of Segmented Influenza A 1 Virus Genome Using a Balance Compensation Strategy) explores the gene expression of IAV after introducing foreign genes in the NS segment using a widely described laboratory-adapted strain such as PR8. The introduction of exogenous genes such as reporter genes into IAV viruses is of crucial relevance to the research community to help in the understanding of different viral processes and the evaluation of countermeasures against Influenza. Unfortunately, one of the main problems regarding the generation of recombinant viruses with heterologous sequences is the modifications of viral properties such as replication and virulence in vivo. The authors tackle this problem by the introduction of mutations in the NCR regions of the NS segments showing a modulation in the vRNA, mRNA, and protein synthesis, followed by characterization in vitro and in vivo of the replication capacity, virulence, and reporter expression; the main conclusion obtained is that a triple mutation of the 3’NCR or NS allows for the introduction of a FLuc reporter which is stable based on Fluc activity, exhibiting similar growth profiles in vitro and 100% lethality after a high dose challenge in vivo.

Overall, the hypothesis and the strategy are clear. The experiments proposed are in line with the answers that the authors should provide. However, one of the main flaws in the work is the segment and strain dependency of the phenomenon observed. It is well established that PR8 is a highly laboratory-adapted strain with an increased tolerance for genome rearrangements and attenuation strategies. Based on this, the relevance of the work is compromised since we cannot establish the applicability of this type of rearrangement in more recent strains such as seasonal H3N2 or pandemic H1N1. For instance, the use of this strategy for the assessment of antiviral efficacy should be performed against current circulating strains, so the relevance of the results presented is conditioned to the tolerance of the rearrangement by different strains and not just PR8.

Furthermore, is this strategy capable of tolerating different HA/NA subtypes? How is the plasticity of the rearrangement studied when other HA/NA combinations of relevance are incorporated? This is in line with the applicability and significance of the strategy that the authors did not address. This reviewer’s opinion is that besides the experimental concerns shown below, the relevance and importance of this strategy need to be studied further. All the mentioned above could improve the originality of the work.

Major comments

Fig 1A shows an empty table. I am not sure if this is an error in the PDF version or the version submitted by the authors. In addition, the scale used in 1C does not allow the view of nuanced differences among groups.

In vitro kinetics (Fig2): It is highly speculative to state an improved replication (‘álmost identical’’) of 38m-Gluc virus compared to PR8-NS Gluc and similar replication when compared with PR8 wt. Results presented are skewed by sharp differences observed at 12h, suggesting differences at time 0, which cannot be ruled out based on the information given. Furthermore, between times 12 and 36, you see (roughly) a three log10 fold increase for PR8 wt, a two-fold increase for NS-Gluc, and just a 1.5 fold increase for the mutants, so I disagree with the authors about the conclusions raised.

What rationale was employed to exclude NS358m from the in vivo experiments in figure 3? As I stated previously, in vitro growth and kinetic data need to be carefully examined. On the other side, NS358m has the higher replication level shown in 2B.

Although the authors provided stability data analyzed using FLuc activity, there is no sequence data about the NS segment and the rest of the IAV genome. What are the nucleotide sequence in the NS segment and the other segments? Does any new nucleotide change appear? Are the three introduced mutations maintained? How does the inclusion of Fluc affect the stability of the HA, for instance, in comparison with the wt virus? All these questions fundamental to understanding the stability of the virus generated remain unanswered. In addition, no information about yield is provided regarding the recombinant viruses. Is the yield comparable to the wt virus?

How many MDL50 related to the PR8 wt is the high dose of PR8-NS358m-Fluc used?

Minor comments

Were samples treated with DNAase for in vitro assays?

Line 140: Please explain briefly how mutations were introduced

Line 392: typo (sepcific)

Fig 2B: Please clarify the nomenclature used for each group and their relationship with the different mutations. The differences in terminology in the text and figure are confusing.

Fig 2D: TCID should say TCID50 in the title

Fig 3C: Baloxivir needs to be corrected
