# Supplementary material for: Expanding the tolerance of segmented Influenza A Virus genome using a balance compensation strategy
Source: PLoS Pathog. 2022 Aug 4;18(8):e1010756. doi: 10.1371/journal.ppat.1010756 (PMC9380948; doi:10.1371/journal.ppat.1010756)
Supplement: Attachment — Submitted filename: Response to reviewer2.docx [file ppat.1010756.s007.docx]

**Point-by-point response to reviewers’ comments**

**Response to Reviewer #2:**

**Point 1.** In the manuscript presented by Zhao et al, the authors researched on the improvement of influenza A segment incorporation when segments are modified for the incorporation of exogenous genes. This is very important for the field because there is an increasing interest for the use of modified influenza A viruses. One major caveat is that these viruses generally exhibit attenuation. The authors have previously demonstrated that one of the major issues relates to an imbalance in the generation of the gene segments. Basically, the modified segment produces less vRNA which in turns leads to attenuation. To overcome this problem, the authors improved the incorporation by mutagenizing the none coding regions (NCR) of a modified (PR8) segment encoding for either firefly or renilla luciferase. They used an in vitro competitive assay in which the WT NCR and the mutant(s) compete. They showed that the mutants were more efficient at producing the reporter, and vRNA and mRNA from the modified segment. However, the in vivo (mice) experiment showed that the improvement was partial; the replication of one of the mutant viruses reached titers comparable to wt but the pathogenesis were not comparable. The mutant virus did not show neither weight loss nor the lethality of wt PR8. The improvement had a more significant impact when the reporter used was larger. The manuscript needs some improvement in terms of writing. Even though the English is clear, there are some colloquial expression that must be avoided. Also, the discussion needs more work. The manuscript presented can be considered for publication after major revisions.

Even though some of the findings seems to address the problem, I think the authors could have dug deeper. In my view, the number of sites and virus used fall short. The authors need to increase the number of viruses and mutation to complement, at least, the in vitro part. I would also try to use other gene segments.

**Response:** We agree with the reviewer on this. The “Balance Compensation” Strategy described in this manuscript is based on the extended studies by others and us, and it just provide a general principle for IAV genome manipulation. But in practice, many factors including the foreign genes, the modified segments and the parental strains should be also considered. The foreign gene dependency of the strategy has been demonstrated in the manuscript clearly, that G3A/C8U but not G3A/U5C/C8U mutations can improve the replication and virulence of recombinant PR8-NS-Gluc, while G3A/U5C/C8U is more suitable than G3A/C8U for directed optimization of recombinant PR8-NS-Fluc.

In addition, we have discussed the segment dependency in Discussion extensively. For example, we propose that U13 to C13 mutation in the 3′ end of the NA can be used as NA-specific enhancement.

In addition, we have successfully generated a recombinant influenza A H3N2/X31 virus carrying Fluc (X31-Fluc). Using X31-Fluc, we have established an in-vivo imaging mouse model of influenza H3N2 virus infection. These data were added in the revised manuscript.

**Point 2.** One major problem with the production of enlarging influenza A segments is that these are usually prone to an increment in the production of defective interfering (DI) particles. The authors did not mention this possibility. I would measure DIs to see if the mutants are less prone to the production of DI particles. This could actually be the reason for the incomplete phenotype showed by the mutants when compared to WT.

**Response:** We have tested the ratio of genome copies to infective virus of the parental PR8-WT, the recombinant PR8-NS-Gluc, PR8-NS^CE1^-Gluc and PR8-NS^CE2^-Gluc viruses. The results clearly demonstrated that PR8-NS^CE1^-Gluc is less prone to the production of DI particles, while PR8-NS^CE2^-Gluc produces as many DIs as PR8-NS-Gluc. These results are in accordance with our data that CE1 could compensate the genome balance and restore the wildtype-like phenotype of PR8-NS-Gluc virus, while CE2 incorporation would cause overcompensation and further attenuation.

**Point 3.** Where the mutant viruses stable? You showed in figure 4C that the PR8-NS358m-Fluc maintained the phenotype, but you do not show any sequencing. The authors need to sequence all the mutant viruses to show stability from the in vitro and in vivo experiments.

**Response：**The genome staibility data were provided in Supporting Information. As shown in Figure S2B, We did observe deletions of the reporter gene for PR8-NS38m-Fluc (PR8-NS^CE1^-Fluc in the revised manuscript) after 3 passages, while the genome of PR8-NS358m-Fluc (PR8-NS^CE2^-Fluc in the revised manuscript) remains stable after 5 passages (Figure S2B). We subsequently sequenced the genome of PR8-NS358m-Fluc (P5) and found one U1G mutation of about 20% frequency (4/21) at the 3’NCR of NA segment and a nonsense A1233U mutation within PA.

**Point 4.** Figure 1. The table looks empty on my end.

**Response:** The figure 1 has been updated accordingly.

**Point 5.** How well conserved are these alleles in the databases.

**Response:** The G3/U5/C8 are highly conserved in all the eight segments of all IAV strains.

**Point 6.** Figure 2B. Please change nomenclature in the figure from NS358m to something like NS3,5,8m or an alternative that helps on differentiating these mutations to mutations on nucleotide position 358.

**Response:** We have updated the terminology to avoid confusion and the figures have been updated accordingly. For example, we use compensatory enhancement 1 (CE1) to refer G3A/C8U mutations, therefore, the NS-Gluc segment carrying G3A/C8U is labeled as NS^CE1^-Gluc, while the recombinant PR8 virus incorporating NS^CE1^-Gluc segment instead of NS is designated as PR8-NS^CE1^-Gluc.

**Point 7.** Supplementary figure 1. Change y-axis legend from mRNA to vRNA

**Response:** We detected the levels of both vRNAs and mRNAs to determine the effects of indicated mutations upon replication and transcription of NS-Gluc segment. We show the vRNA data in main text, and mRNA data in supporting information.

**Point 8.** Line 169-170. “Over-compensation would on the other hand disrupt the balance of the multiple segments, which also results in attenuation”. You are assuming this is the main mechanism by which NS358m-Gluc is less efficient in vivo. But it can be any other, like cells being more sensitive to RNA detection, stronger IFN response, etc. Which at the end of the day they are all related but you guys do not show any on this respect. Since this is still speculation, I suggest to either remove the interpretation or move it to discussion and address as speculative.

**Response:** We moved the interpretation to Discussion. Of note, regarding original NS-Gluc, as we discussed in Introduction, the impaired replication of segment NS-Gluc may results in decreased NS1 expression, which is usually related to stronger IFN response and signaling. However, in the case of NS358m-Gluc, the overcompensation of segment replication would cause over-expression of NS1, which is supposed to help the virus to antagonize cellular IFN response and signaling.

**Point 9.** Line 209 live imaging remains a mission impossible. There is no impossible for science. We just don’t know yet.

**Response:** We have revised the sentence as “live imaging in the PR8-NS^CE1^-Gluc infected mice was not successful after several rounds of attempt (data not shown).”.

**Point 10.** As Fig 4B shows, the replication of the original NS-Fluc is impaired much worse compared to NS- Gluc, not to mention the natural NS. I think there could be more proper ways to write about the results.

**Response:** We have revised the description as “the replication of NS-Fluc was greatly impaired, with a signal roughly 0.2% of the parental NS, which was even worse than NS-Gluc (10% of the parental NS)”

**Point 11.** Line 275. Please, find a different word for “Encouragingly”.

**Response:** We have revised the Discussion extensively.

**Point 12.** Line 305-306. “…may contribute a lot”. Please, reword.

**Response:** We have revised the Discussion extensively.
